# Double-stranded RNA-activated protein kinase PKR of fishes and amphibians: Varying the number of double-stranded RNA binding domains and lineage-specific duplications

**DOI:** 10.1186/1741-7007-6-12

**Published:** 2008-03-03

**Authors:** Stefan Rothenburg, Nikolaus Deigendesch, Madhusudan Dey, Thomas E Dever, Loubna Tazi

**Affiliations:** 1 Laboratory of Gene Regulation and Development, National Institute of Child Health and Human Development, National Institutes of Health, Bethesda, MD 20892, USA; 2 Institute for Immunology, University Hospital Eppendorf, 20246 Hamburg, Germany; 3 Department of Biology, Brigham Young University, Provo, UT 84602, USA

## Abstract

**Background:**

Double-stranded (ds) RNA, generated during viral infection, binds and activates the mammalian anti-viral protein kinase PKR, which phosphorylates the translation initiation factor eIF2α leading to the general inhibition of protein synthesis. Although PKR-like activity has been described in fish cells, the responsible enzymes eluded molecular characterization until the recent discovery of goldfish and zebrafish PKZ, which contain Z-DNA-binding domains instead of dsRNA-binding domains (dsRBDs). Fish and amphibian PKR genes have not been described so far.

**Results:**

Here we report the cloning and identification of 13 PKR genes from 8 teleost fish and amphibian species, including zebrafish, demonstrating the coexistence of PKR and PKZ in this latter species. Analyses of their genomic organization revealed up to three tandemly arrayed PKR genes, which are arranged in head-to-tail orientation. At least five duplications occurred independently in fish and amphibian lineages. Phylogenetic analyses reveal that the kinase domains of fish PKR genes are more closely related to those of fish PKZ than to the PKR kinase domains of other vertebrate species. The duplication leading to fish PKR and PKZ genes occurred early during teleost fish evolution after the divergence of the tetrapod lineage. While two dsRBDs are found in mammalian and amphibian PKR, one, two or three dsRBDs are present in fish PKR. In zebrafish, both PKR and PKZ were strongly upregulated after immunostimulation with some tissue-specific expression differences. Using genetic and biochemical assays we demonstrate that both zebrafish PKR and PKZ can phosphorylate eIF2α in yeast.

**Conclusion:**

Considering the important role for PKR in host defense against viruses, the independent duplication and fixation of PKR genes in different lineages probably provided selective advantages by leading to the recognition of an extended spectrum of viral nucleic acid structures, including both dsRNA and Z-DNA/RNA, and perhaps by altering sensitivity to viral PKR inhibitors. Further implications of our findings for the evolution of the PKR family and for studying PKR/PKZ interactions with viral gene products and their roles in viral infections are discussed.

## Background

The double-stranded (ds) RNA-activated protein kinase PKR (eIF2aK2) is an integral component of the innate immune response (reviewed in [1-3]). In mammals PKR, which contains two N-terminal dsRNA-binding domains (dsRBDs) [4], is constitutively expressed at moderate levels in most cells types and can be transcriptionally induced approximately five-fold after immunostimulation by interferons or dsRNA. PKR is a first line defense molecule against viral infection. Immediately after infection or early during replication or transcription of viral genes, even before the interferon response kicks in, viral dsRNA can activate PKR. Elevated levels of PKR after interferon induction sensitizes cells to react even more strongly to viral pathogens leading to a general inhibition of protein synthesis and potentially to apoptosis.

PKR has been shown to be crucial for the host response against a variety of viral pathogens. An important role for PKR in the antiviral response is further supported by the finding that many viruses evolved inhibitors of PKR (reviewed in [1,3,5]). In one model for PKR activation, the two dsRBDs found in the amino-terminal part of mammalian and avian PKR are thought to fold back onto the kinase domain thereby inhibiting dimerization and kinase activity [6]. Upon binding of dsRNA, this autoinhibition is relieved facilitating the dimerization of two PKR molecules. This dimerization is mediated by both the N-terminal dsRBDs as well as by residues of the kinase domain and is a prerequisite for the activation of PKR which is accompanied by the trans-autophosphorylation of many serine and threonine residues [7-11].

The best-characterized substrate of PKR is the α subunit of eukaryotic translation initiation factor 2 (eIF2), which is phosphorylated at Ser51. Phosphorylation of eIF2α is one of the best-understood mechanisms enabling cells to rapidly alter protein production in response to environmental stimuli (reviewed in [12]). eIF2 consists of three subunits, α, β and γ. When bound to GTP, eIF2 forms a ternary complex with initiator methionyl-tRNA, which is essential for cap-dependent translation initiation. Binding of this complex to the 40S ribosomal subunit generates a 43S preinitiation complex that binds mRNA and scans to identify a start codon. Following base-pairing of the anticodon of the tRNA to an initiation codon, scanning is halted, and the 60S subunit joins. This coincides with the hydrolysis of bound GTP to GDP and dissociation of eIF2. In order to allow a new round of translation initiation, the GDP bound to eIF2 must be exchanged for GTP by the guanine nucleotide exchange factor eIF2B. Phosphorylation of the eIF2α on Ser51 converts eIF2 into a competitive inhibitor of eIF2B, resulting in decreased levels of GTP-bound eIF2 and leading to the general inhibition of translational initiation [13,14]. However, a small subset of mRNAs is translated more efficiently after eIF2α phosphorylation. The mRNAs encoding the transcription factors GCN4 in yeast (reviewed in [15]) and ATF4 in vertebrates [16] contain short upstream open reading frames (uORFs), which inhibit the efficient translation of the genuine ORFs when the ternary complex is abundant [16]. When the levels of ternary complex are reduced after eIF2α phosphorylation the genuine ORFs are translated more efficiently.

A family of protein kinases has been identified that mediate translational regulation through phosphorylation of eIF2α. These kinases all share phylogenetically closely related kinase domains that are linked to unique regulatory domains that determine the mode of kinase activation (reviewed in [17]). In addition to PKR, four other eIF2α kinases have been identified in vertebrates: the heme-regulated inhibitor kinase (HRI/eIF2aK1) is activated under conditions of heme deprivation and arsenite exposure, the PKR-like endoplasmatic reticulum kinase (PERK/PEK/eIF2aK3) is activated by unfolded proteins in the ER, and GCN2 (eIF2ak4) is activated by uncharged tRNA and thus senses amino acid starvation.

The fifth and most recently discovered member of the eIF2α kinase family is PKZ, which was cloned from goldfish, zebrafish and Atlantic salmon [18-20]. The kinase domain of PKZ is most closely related to PKR, however it contains two Z-DNA binding domains, called Zα domains, in the N-terminus instead of dsRBDs, as found in PKR [18,19]. The Z-conformation is an alternative higher-energy form that can be adopted by double-stranded nucleic acids. In Z-DNA and Z-RNA the (deoxy)ribose backbone forms a left-handed zigzag helix, as opposed to the smooth right-handed helix in B-DNA and A-RNA [21,22]. In cells, formation of Z-DNA is induced by negative supercoiling, generated by moving RNA-polymerases during transcription, and can modulate promoter activity probably by alternating the local architecture of promoter regions and nucleosome positioning [23,24].

Zα domains specifically bind, stabilize and induce Z-DNA and Z-RNA [22,25,26]. They adopt a helix turn helix conformation [27,28] that is structurally unrelated to the α-β-β-β-α fold adopted by dsRBDs [29]. Until now, Zα domains have only been identified in three cellular Z-form binding proteins (ZBPs): the RNA-editing enzyme ADAR1 (reviewed in [1]), which also contains three dsRBDs, Z-DNA binding protein 1 (ZBP1/DLM1) [30] and PKZ. In all three proteins two Zα domains are found at the amino-terminus and are separated by a linker that varies in size between 16 and 91 amino acids [19]. In addition, expression of these cellular ZBPs are highly induced upon immunostimulation, and the Zα domains determine the subcellular localization of the proteins [26,31,32].

Interestingly, the poxvirus protein E3L, one of the best studied PKR inhibitors, contains both a Zα domain and a dsRBD [33], combining the two nucleic acid binding domains found in PKZ and PKR. Both domains in E3L have been found to be essential for Vaccinia virus pathogenesis in a mouse model [34]. The importance of Z-form binding is further indicated by the finding that single point mutations in the Zα-domain that prevented Z-DNA binding greatly reduced virus pathogenicity. Remarkably, pathogenicity was fully restored by substitution of the Zα domain of E3L with that of ADAR1 or ZBP1 despite a low sequence identity of about 25% (see [35]).

When transfected into mammalian cells, PKZ inhibited reporter gene expression to comparable levels as previously observed for PKR [19]. However, an important difference is that a kinase inactivating point mutation in PKR (K296R) had stimulating effects on reporter protein expression (two- to six-fold) [36,37], while kinase inactivated PKZ (K199R) had neither stimulatory nor inhibitory effects [19]. These findings indicate that endogenous PKR, which is probably activated in every transfection, can be inhibited in a dominant negative fashion only by inactive forms of PKR but not by PKZ. Moreover, these results suggest that dsRBDs and Zα domains have different binding partners or activators. Identification of PKZ, a PKR homolog in fish possessing different nucleic acid binding domains, is striking and may reflect adaptation to the different viruses challenging these species.

Hitherto PKR has only been identified in mammals and chickens. However, similar activity has also been described in fish cells but the enzymes responsible eluded molecular cloning for a long time [38,39]. The identification and characterization of genes that are important in the immune response in poikilotherm vertebrates is of considerable interest because their pathogens have seriously decimated both wild-type and farm-raised populations leading to significant ecological and economical losses [40]. In this report, we characterize PKR genes of fish and amphibians, demonstrating the coexistence of PKR and PKZ in at least one fish species, and we identify lineage-specific duplications of PKR genes. Our findings support the notion that PKR and PKZ play similar but not identical roles in host defense and open the door to investigate the role of PKR in the innate immune response of poikilotherms and in the interplay with pathogens.

## Results

### Cloning of three PKR genes from *Tetraodon nigroviridis*

The sequences of two partial PKR genes representing parts of the kinase domains from the freshwater pufferfish *T. nigroviridis *have been reported [41]. Since these sequences are incomplete and lack 5' and 3' regions it was not clear whether these genes contain dsRBDs, Z-DNA binding domains (Zα domains) or other regulatory domains.

**Figure 1:**
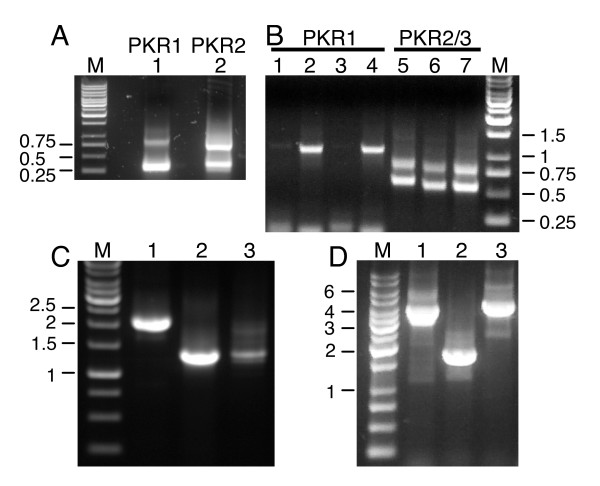
**PCR for cloning of *T. nigroviridis *PKR genes**. (A) Results for 5' RACE PCRs with *T. nigroviridis *cDNA are shown for primers specific for PKR1 (lane 1) and PKR2 (lane 2). M denotes the 1 kb marker. Fragments are labeled in kb. (B) shows the results of 3' RACE PCR using four different forward primers used in primary and nested PCRs for PKR1 (lanes 1–4) and three different forward primers combinations for PKR2 and PKR3 (lanes 5–7). The smaller fragment represents PKR2 and the larger one represents PKR3. (C) PCR products are shown using primers covering the complete open reading frames of PKR1 (lane 1), PKR2 (lane 2) and PKR3 (lane 3). (D) PCR reactions of overlapping regions were performed with genomic DNA to elucidate the genomic organization of PKR1. Lanes 1 and 2 show the PCR products obtained with primers spanning the region between the 5' untranslated region of PKR1 and exon 15 and exon 14 and the 3' untranslated region of exon 19, respectively. PCR product shown in lane 3 was obtained with primers covering exon 17 of PKR1 and exon 7 of PKR2. PCR products were cloned and completely sequenced.

Because the draft *T. nigroviridis *genome sequence was not of high enough quality for reliable gene prediction, we performed 5' and 3' rapid amplification of cDNA ends (RACE) experiments in order to obtain the complete gene sequences. Since both PKR and PKZ genes are inducible by immunostimulation, we treated a *T. nigroviridis *fish with polyinosinic-polycytidylic acid (poly(I:C)) for 12 h to induce expression. Different freshwater pufferfish species often look similar and reliable discrimination between these species can only be achieved by molecular techniques [42]. To confirm the species of our pufferfish, a 306 bp fragment from the cytochrome B (cytb) gene was cloned and sequenced. The obtained sequence was 100% identical to 12 *T. nigroviridis *cytb gene sequences deposited in the database (e.g. accession number AJ248568). The closest related sequences were from *T. fluviatilis*, displaying 97% identity. These results demonstrate that our pufferfish belongs to the species *T. nigroviridis*. We designed primers from the available PKR1 and PKR2 sequences and performed 5' and 3' RACE polymerase chain reaction (PCR). For 5' RACE PCR of PKR1 and PKR2, 800 and 700 bp bands were observed, respectively (Figure 1A). The longest fragment obtained for PKR1 contained 872 bp with the first ATG at position +60 (relative to the presumptive transcription start site). An in-frame stop codon at position +55 preceded this AUG codon, which initiated an ORF that extended through the entire sequence. The 5' RACE for PKR2 yielded a 710 bp fragment with a start codon at position +29, that was preceded by an in-frame stop codon at position +18. The two lower molecular weight bands represent non-specific PCR products.

Different primers were used for 3' RACE PCR of PKR1, which all yielded a single major band that was amplified to different extents (Figure 1B, lanes 1–4). For PKR2, two major bands were amplified (Figure 1B, lanes 5–7). Cloning of the PCR products showed that they all contained poly-A tails. The two fragments obtained with PKR2-specific primers displayed 87% identity in their deduced coding sequences. Since these two sequences represent two closely related PKR genes, we are referring to them as PKR2 and PKR3 in the following paragraphs.

**Figure 2:**
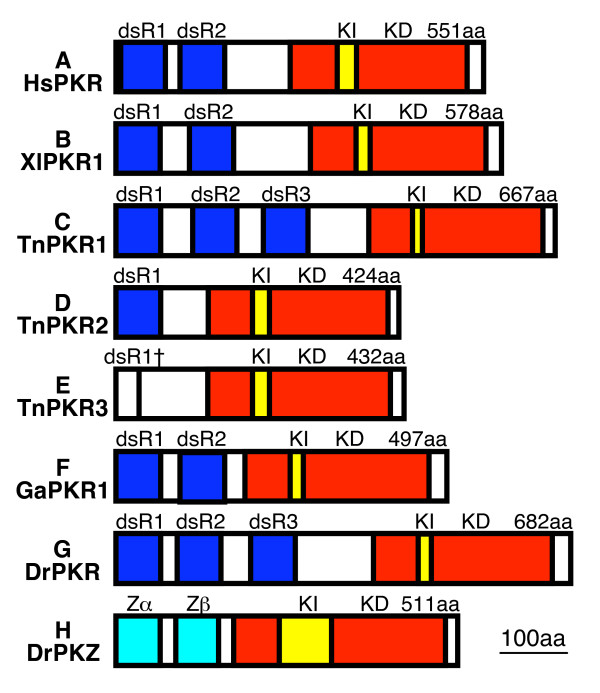
**Schematic presentation of the domain organization of PKR and PKZ genes**. The domain organization of PKR and PKZ genes from (A) *H. sapiens *(Hs), (B) *X. laevis *(Xl), (C)-(E) *T. nigroviridis *(Tn), (F) *G. aculeatus *(Ga) and (G), (H) *D. rerio *(Dr) are shown in the following colors: dsRNA binding domains (dsR), dark blue; Z-DNA binding domains (Zα), light blue; kinase domains (KD), red; kinase inserts (KI), yellow. Total length of deduced open reading frames in amino acids (aa) is indicated above the schematics.

Reverse transcription (RT)-PCR covering the complete ORF using primers derived from the sequences obtained from 5'- and 3'-RACE PCR, showed products of about 2.1 kb for PKR1 and about 1.2 kb for PKR2 and PKR3 (Figure 1C). Cloning and sequencing of the fragments showed that PKR1 contains an ORF of 2001 bp. Three dsRBDs (dsRB1-3) were identified in the N-terminal region using the conserved domain database [43] (Figure 2C). While dsRBD1 and dsRBD3 showed highly significant expected values (*E*-value) of 3 × 10^-13 ^and 1 × 10^-11^, respectively, the *E*-value for dsRBD2 was only 0.013. PKR2 and PKR3 contain 1272 and 1296 bp ORFs, respectively. Since only a single cDNA species was cloned for the 5' RACE of PKR2, the same forward primer originating from the 5' RACE of PKR2 was used for PKR2 and PKR3. The weaker band intensity of PKR3 is probably not a result of lower expression, as amplification worked comparably well for PKR2 and PKR3 in the 3' RACE, but reflects that the primer used has two mismatches to PKR3 in the middle of the primer and amplification was thus probably less efficient. Only one complete dsRBD was identified in PKR2 (*E*-value = 2 × 10^-14^), while only a partial dsRBD, which contains only the N-terminal portion of the dsRBD, is present in PKR3 (Figures 2D and 2E). TnPKR2 dsRBD is most closely related to TnPKR1 dsRBD1 (80% amino acid identity), followed by dsRBD3 (40% identity) and dsRBD2 (28% identity). Amino acid identity in the kinase domain of PKR1 with PKR2 and PKR3 is 49% and 49.4 %, respectively, while that of PKR2 and PKR3 is 88.5%. A muliple sequence alignment of the dsRBDs are shown in Additional file 1.

### Cloning and expression analysis of a zebrafish PKR gene

**Figure 3:**
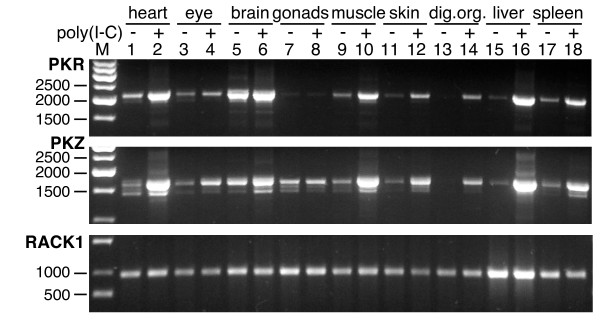
**Comparison of expression patterns of zebrafish PKR and PKZ after induction with poly(I:C)**. PCRs were performed with cDNA prepared from the indicated tissues with primers covering the complete ORFs of zebrafish PKR (upper panel), PKZ (middle panel) or RACK1, the latter of which is constitutively expressed and served as control (lower panel). Zebrafish were either treated with poly(I:C) (indicated by plus) or with PBS (minus).

We searched the genomic zebrafish (*Danio rerio*) database (see Methods) with the amino acid sequence of TnPKR1 in order to identify a PKR gene using protein query versus translated database (tblastn). We identified sequences with apparent homology to the 5' and 3' regions of TnPKR1 that were present in two different contigs. To determine whether the identified regions belong to a single gene, we designed primers from the predicted 5' and 3' untranslated regions, performed RT-PCR and successfully amplified fragments of about 2.1 kb from various organs (Figure 3, upper panel). Cloning of these fragments revealed an ORF of 2046 bp. Like TnPKR1, DrPKR contains three dsRBD (Figure 2G). Two major alleles, PKRa and PKRb, varying at four amino acid positions (V91I; P172Q; L355S; M615V) were identified.

In order to analyze whether drPKR displays a similar expression profile to the previously identified PKR relative PKZ and to investigate whether it is also inducible by poly(I:C), we performed RT-PCR with previously prepared RNA from different organs of zebrafish that were either treated with poly(I:C) or mock-treated with phosphate buffered saline (PBS) for 12 h [19]. Primers were used that cover the complete ORFs of PKR and PKZ. The housekeeping gene RACK1 was used as a control. As observed for PKZ, PKR was expressed at low to moderate levels in most untreated tissues expect for brain, where a strong signal was observed (Figure 3). In the poly(I:C) treated animals, PKR was highly induced in heart, muscle, skin, digestive organs, liver and spleen. In these organs PKR showed comparable induction to PKZ. No or only moderate induction of PKR was observed in the eye, brain and gonads. The most striking expression differences between PKR and PKZ were observed in the gonads, where PKR is almost absent and PKZ is moderately expressed but not induced, and in the brain, where PKR appears to be expressed at higher basal levels. The different bands observed for PKZ are a result of previously described different splice products [19]. The occurrence of gene sequences in the database for expressed sequence tags (dbEST) can be used to roughly estimate the abundance of transcripts. Currently, seven *D. rerio *PKZ EST clones were found in the dbEST database, while none was identified for PKR.

### Identification of PKR genes from other fish species

Using the cloned fish PKR genes as probes, we used tblastn to search dbEST and genomic databases in order to identify more PKR genes. We identified two PKR genes in the genome of the fugu fish (*Takifugu rubripes*). They are present in scaffold 459 and are separated by approximately 3 kb. Owing to the relatively high sequence conservation in the kinase domains, we were able to identify these with a high level of confidence. On the amino acid level, the kinase domains of TnPKR1 and TnPKR2 are around 71% and 66%, identical to those of TrPKR1 and TrPKR2, respectively. We identified two putative dsRBDs, each encoded by two exons, in the 5' region of each *T. rubripes *PKR gene. Owing to the poorer sequence conservation outside the kinase domain, and the lack of expression analysis and ESTs, we could not reliably determine the exact structure of these genes.

We identified nine overlapping, high-quality EST sequences covering the complete 497 amino acids predicted ORF of a single PKR gene from the three-spined stickleback (*Gasterosteus aculeatus*). Two predicted dsRBDs are present in the N-terminus (Figure 2F). The segment linking the 3' end of dsRBD2 and the 5' end of the kinase domain is remarkably small comprising only 29 amino acids. The complete gene was also identified in the *G. aculeatus *draft genome sequence on contig 5174. The kinase domain of a second PKR gene, whose expression is not supported by ESTs, was identified approximately 12 kb upstream of GaPKR1.

Searching the genome of the Japanese medaka (*Oryzias latipes*), we identified one PKR gene in the contig BAAF02021246. As for the TrPKR genes, we were able to identify the kinase domain with a high level of confidence but could not precisely define the rest of the gene or the exact borders of the exons encoding two putative dsRBD present in the 5' region.

The kinase domain of the fathead minnow (*Pimephales promelas*) was reconstructed from four overlapping EST sequences and five EST sequences were identified that probably encode for two PKR dsRBDs. However, as the two constructed contigs did not overlap, it is presently unknown whether the dsRBDs and the kinase domain sequences belong to the same gene.

### Cloning and identification of PKR genes from additional vertebrates

We screened dbEST for PKR genes from additional organisms. ESTs from the putative 5' and 3' end of a *Xenopus laevis *PKR gene were identified. We designed primers from the predicted untranslated regions of this gene, performed RT-PCR and cloned and sequenced XlPKR1 (not shown). The predicted 1737 bp ORF encodes for a 65 kD PKR ortholog, which contains two dsRBDs (Figure 2B). Eleven ESTs were identified for XlPKR1. Two additional *X. laevis *ESTs, representing parts of the kinase domain and showing 65–80% identity on the protein level compared with XlPKR1, were identified using tblastn. These ESTs probably represent a second PKR gene from this species.

**Figure 4:**
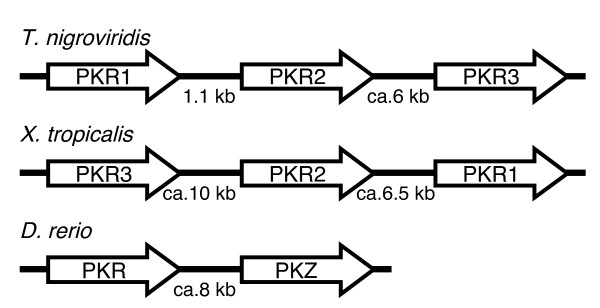
**Genomic arrangement of tandemly arranged PKR and PKZ genes in *T. nigroviridis*, *X. tropicalis *and *D. rerio***. The genomic arrangement and relative orientation of three PKR genes in *T. nigroviridis *and *X. tropicalis *and PKR and PKZ in *D. rerio *are shown. Arrows indicate 5' to 3' orientation of genes. The distance or approximate sizes of intergenic regions are indicated.

Using XlPKR1 as a probe, we searched for PKR ESTs from the related species *X. tropicalis *and identified 22 ESTs. Since these ESTs are overlapping and span the complete predicted ORF, we were able to '*in silico*' clone this gene, termed XtPKR1. Like XlPKR1, XtPKR1 contains two dsRBDs and displays 73% amino acid identity with the former. In addition, we identified eight ESTs from two closely related genes, which we named XtPKR2 and XtPKR3. Combining the sequences of these ESTs and the information from the draft genome of *X. tropicalis*, we were able to identify the complete kinase domains of these additional PKR genes. The three *X. tropicalis *PKR genes are tandemly arranged in head-to-tail (parallel) orientation on the same contig, in the order of PKR3, PKR2 and PKR1, with respect to 5' to 3' orientation of the genes, and are separated by approximately 10 and 6.5 kb, respectively (Figure 4). Sequences coding for putative dsRBDs were also identified 5' to the kinase domains of XtPKR2 and XtPKR3 and are partly represented in one EST for each gene. Owing to gaps in the genomic sequence and a high level of sequence variability outside the kinase domains, the determination of the exact gene organization was not possible.

In an attempt to reconstruct the phylogeny of the PKR gene family, we aimed to identify mammalian PKR sequences in addition to those already deposited in GenBank. Owing to the relatively high conservation of sequences and exon-intron boundaries in the PKR genes within the primate family, we successfully identified the PKR gene of the rhesus monkey (*Macaca mulatta *(Mam)) using tblastn searches of the draft *M. mulatta *genome sequence. The coding exons of MamPKR were found in seven contigs that ranged in size between 1 and 50 kb. The obtained sequence is identical to the recently released MamPKR sequence (accession number NP_001077417), except for an Asn to Arg substitution at position 384, which most likely represent different alleles. The sequence identity of MamPKR is highest when compared with African green monkey (92%), human (80%) and chimpanzee (80%).

The putative PKR sequences of the monotreme *Ornithorhynchus anatinus *(platypus) and the marsupial *Monodelphis domestica *(gray short-tailed opossum), which were predicted using automatic gene prediction approaches, are found in the Ensembl database [44]. Three alternative and likely mutually exclusive predicted transcripts are deposited for the PKR gene of each species. Careful examination of the suggested transcripts and multiple alignments with other PKR sequences indicate that ENSMODT00000030492 (for *M. domestica*) and ENSOANT00000006231 (for *O. anatinus*) contain the correct kinase domains that we used for further phylogenetic analyses.

### Fish PKR genes are more closely related to fish PKZ than to PKR genes of other vertebrates

**Figure 5:**
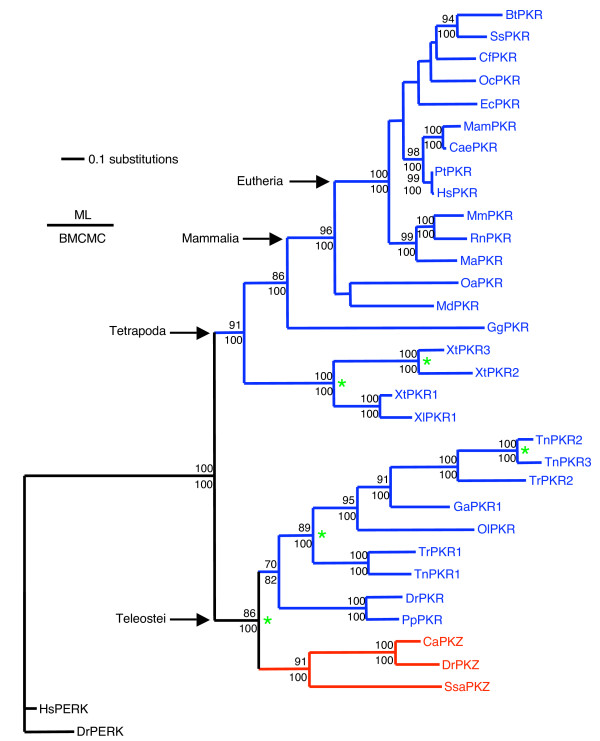
**Phylogenetic relationship of PKR and PKZ genes**. The phylogenetic tree was constructed from the kinase domains of PKR (blue branches) and PKZ (red branches), omitting the KI, using maximum likelihood and BMCMC approaches. Both analyses resulted in the same tree topology. Significant bootstrap values above 70 (for maximum likelihood analysis) and significant posterior probabilities converted to percentages above 95 (for Bayesian analysis) are shown above and below the branches, respectively. Asterisks denote evident duplication events. Human and zebrafish PERK were used as outgroups for rooting the phylogenetic tree. The following abbreviations were used: Bt, *Bos taurus *(cattle); Ss, *Sus scrofa *(pig); Cf, *Canis familiaris *(dog); Oc, *Oryctolagus cuniculus *(European rabbit); Ec, *Equus caballus *(horse); Mam, *Macaca mulatta *(Rhesus macaque); Cae, *Chlorocebus aethiops *(African green monkey); Pt, *Pan troglodytes *(chimpanzee); Hs, *Homo sapiens *(human); Mm, *Mus musculus *(house mouse); Rn, *Rattus norvegicus *(Norway rat); Ma, *Mesocricetus auratus *(golden hamster); Oa, *Ornithorhynchus anatinus *(platypus); Md, *Monodelphis domestica *(gray short-tailed opossum); Gg, *Gallus gallus *(chicken); Xt, *Xenopus tropicalis *(western clawed frog); Xl, *Xenopus laevis *(African clawed frog); Tn, *Tetraodon nigroviridis *(green spotted pufferfish); Tr, *Takifugu rubripes *(torafugu); Ga, *Gasterosteus aculeatus *(three spined stickleback); Ol, *Oryzias latipes *(Japanese medaka); Dr, *Danio rerio *(zebrafish); Pp, *Pimephales promelas *(fathead minnow); Ca, *Carassius auratus *(goldfish); Ssa, *Salmo salar *(Atlantic salmon).

We have previously shown that the kinase domain of fish PKZ is more closely related to that of mammalian and chicken PKR than to the other eIF2α kinases [19]. In order to determine the phylogenetic relationship within the extended PKR/PKZ family we performed phylogenetic analyses with the sequences of the kinase domains, as described in Methods. Kinase domains of human and zebrafish PERK, which is the closest relative of PKR/PKZ [19], were used as outgroups for rooting the phylogenetic trees. These outgroups confirmed the stability of the topology of the trees. A comparable topology of the PKR/PKZ clade was obtained when the other two eIF2α kinases GCN2 and HRI were included in our phylogenetic analyses (data not shown). Both phylogenetic analyses resulted in the same tree topology (Figure 5). Significant bootstrap values above 70 (for maximum likelihood analysis) and significant posterior probabilities converted to percentages above 95 (for Bayesian analysis) are shown above and below the branches, respectively.

Two major clades were recovered with high statistical support (Figure 5). While in one clade the fish PKR genes were nested with the PKZ genes, the other clade contained the PKR genes of all other vertebrates. This demonstrates that the fish PKR genes are more closely related to PKZ than to PKR of the other vertebrates. The pufferfish TnPKR1 and TrPKR1 sequences are nested with one another as well as TrPKR2 with TnPKR2 and TnPKR3. Within the fish PKR/PKZ clade, the occurrence of three independent duplication events is evident (marked with asterisks in Figure 5), the first of which resulted in the split into PKZ and PKR genes. The other major clade approximately recapitulates vertebrate evolution, with the *Xenopus sp*. sequences branching off in a basal node. A close kinship of the three *X. tropicalis *PKR genes is observed which probably reflects two gene duplication events (marked with asterisks in Figure 5). Chicken (Gg) PKR branches off before the mammalian PKR genes. Within the mammalian clade, platypus and opossum PKR genes are basal to the PKR genes of the placental mammals. It is important to point out that PKR genes of fish, for example the three PKR genes of *T. nigroviridis*, and amphibians, for example the three PKR genes of *X. tropicalis*, while sharing a common ancestor, arose from independent gene duplications and should therefore be viewed as paralogs, not orthologs.

### Lineage-specific amino acid substitution in the kinase domains of PKR and PKZ

**Figure 6:**
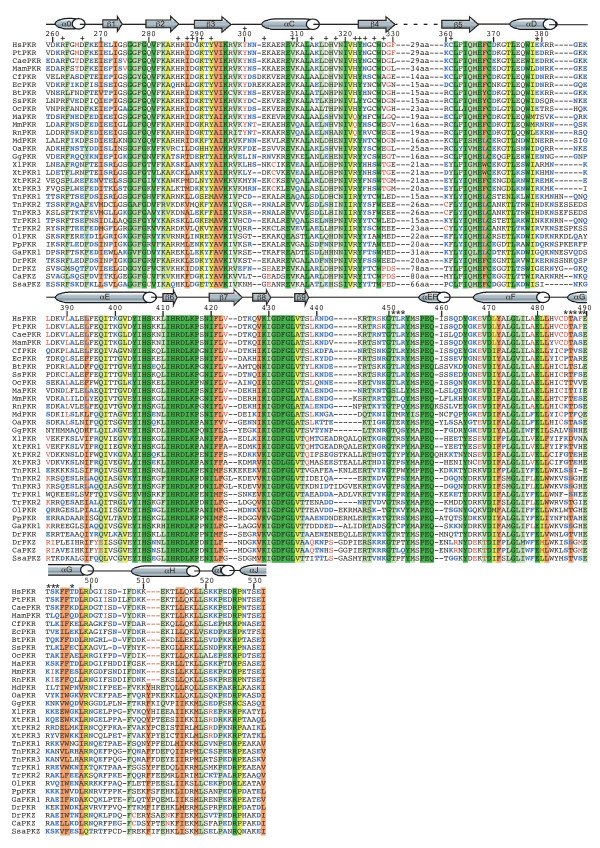
**Multiple sequence alignment of the kinase domains of PKR and PKZ genes from various species**. Multiple sequence alignment of the kinase domains PKR and PKZ from the indicated species (abbreviations are explained in Figure 5 legend) was performed using MUSCLE [64]. Secondary structure elements as reported for human PKR [10] as well as numbering of residues relative to human PKR are shown above the sequences. Residues involved in PKR inter-dimer contacts (pluses) and eIF2α recognition (asterisks) are marked above the sequences. Residues or deletions that show evidence for convergent evolution (blue) or are lineage specific (red) are colored. The backgrounds of residues that are highly conserved are colored as follows: 100% conservation, dark green; 90–99% conservation, light green; 80–89% conservation, yellow; conservation of functionally conserved residues, salmon pink.

Multiple sequence alignment of the kinase domains of PKR and PKZ genes (Figure 6; numbering of amino acid position is shown relative to human PKR), in combination with the phylogenetic tree, reveal a number of lineage-specific amino acid substitutions that are not found in genes of other lineages (highlighted in red in Figure 6). Some examples are mentioned hereafter. Residues specific for the Tetrapoda lineage are Gly329 and Glu468, the latter of which replaced a Lys that is found in fish PKR and PKZ as well as in PERK. Mammalia-specific residues are Leu390, Val423, Leu439 and Cys485. Eutheria-specific residues are Tyr300 and Ala479. Leu386, Val484, Asp486 and Ile503 are specific for primates. Residues found specifically in PKZ genes are Pro328, Arg386, Glu467 and Thr469. A three amino acid deletion between residues 510 and 511 is specific for the eutheria lineage.

A highly acidic kinase insert (KI) linking kinase subdomains IV and V is specific for eIF2α kinases and is highly divergent in length. A striking difference in the length of the KI is observed between PKZ and PKR sequences, which ranges from 14–34 residues for PKR and 66–80 residues for PKZ (see Additional file 2). This insert contains a high proportion of Ser/Thr residues that are targets for autophosphorylation. Taken together, the four amino acids Asp, Glu, Ser and Thr account for an average of 56.1% and 54.7% of all residues of the KI of PKR and PKZ, respectively.

### Convergent evolution of PKR/PKZ genes at multiple sites

Evidence for the convergent evolution of PKR genes, if the multiple sequence alignment (Figure 6) and the phylogenetic tree (Figure 5) are taken into account, is found at multiple positions. Examples where the same amino acid substitution apparently evolved independently (highlighted in blue in Figure 6), include position 261 (independent lysine, alanine, glutamate or serine substitutions), position 290 (independent glycine, lysine, asparagine, aspartate or glutamate substitutions), position 314 (independent lysine, arginine, aspartate, glutamate or threonine substitutions), position 400 (four independent lysine substitutions), position 449 (independent lysine, arginine, isoleucine and threonine substitutions), position 493 (independent lysine, glutamate or glutamine substitutions) and position 524 (independent glutamate, lysine or alanine substitutions). Of note is also the independent emergence of a tyrosine at position 363 in the fish PKR lineage and in XtPKR2 and OaPKR because it affects the sequence of the LFIQMEF motif that has been identified as important for eIF2α kinase activity [45].

### Only a few residues involved in PKR dimer contact or substrate recognition are highly conserved

Dimerization of eIF2α kinases that is partly mediated by residues in the kinase domain (dimer contacts as observed in the crystal structure are marked with plus signs (+) in Figure 6) is essential for kinase activity [10]. A salt bridge formed between Arg262 on one protomer and Asp266 on the other is important for dimerization of hsPKR [46]. While residues making this salt bridge are present in all but platypus (Oa) mammalian PKR genes, a predicted functional salt bridge is only found in 7 out of 17 non-mammalian PKR/PKZ genes. Another important contact as revealed by site directed mutagenesis is made with residues Asp289 and Tyr293 on one protomer with Tyr323 on the other protomer forming an H-bond triad. While Tyr323 is conserved in all PKR/PKZ genes, a tyrosine is found at position 293 in 27 out of 31 genes and an acidic residue at position 289 in 22 out of 31 sequences. Of the other contacts, only Val309 is highly conserved.

Residues that contact the substrate eIF2α are marked with asterisks (Figure 6). Of these only Thr(Ser)451, Thr(Ser)487 and Glu490 are highly conserved.

### Similar genomic organization of PKR and PKZ genes

**Figure 7:**
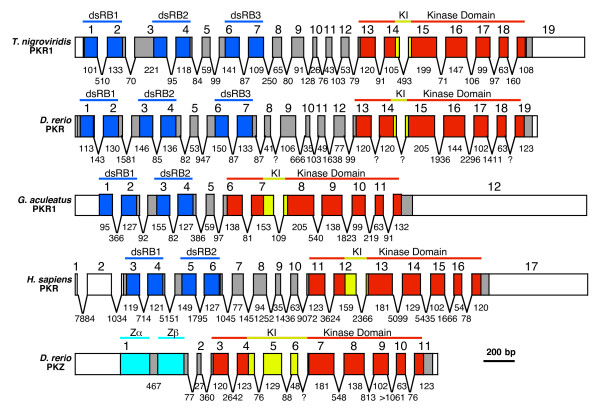
**Exon/intron organization of PKR and PKZ genes**. The exon/intron structure of *T. nigroviridis *PKR1, *D. rerio *PKR and PKZ, *G. aculeatus *PKR and *H. sapiens *PKR (as reported [47]). Untranslated regions of exons are represented as white boxes, while colored and gray boxes denote exons of ORFs. Exonic parts encoding dsRBDs (dark blue), Z-DNA binding domains (Zα; light blue), kinase domains (red) and KIs (yellow) are colored. Exons are drawn to scale. Lengths (in bp) of exonic parts contributing to the ORFs are shown below the exons. Lengths (in bp) of introns are shown in between exons. Question marks indicate intron sizes of unknown length.

Owing to large gaps or large stretches of unknown sequences, the draft *T. nigroviridis *genome sequence was of limited help in the cloning process and in determining the genomic organization of *T. nigroviridis *PKR genes. Partial sequences of PKR1 and PKR2 were identified in the same contig in the genomic sequence, indicating close proximity of these genes. However, the last 189 nucleotides of the 3' coding sequence of PKR1 were missing in the draft genome as well as the first part of the 5' coding region of PKR2. In order to better understand the genomic organization of the PKR genes, we performed PCR from genomic DNA. We were successful in amplifying three overlapping fragments covering the complete PKR1 gene, the intergenic region between PKR1 and PKR2 and the first half of PKR2 (Figure 1D). This 8.3 kb region was completely sequenced. PKR1 consists of 19 exons covering approximately 5 kb (Figure 7). The overall genomic organization is remarkably similar to that of human PKR [47]. Each dsRBD is encoded by two exons. The kinase domain is encoded by seven exons. The shortest exon (exon 10) of tnPKR1 is only 26 bp long. The transcription start site of TnPKR2 was identified only 1.1 kb 3' of exon 19 of TnPKR1 (Figure 4). In the current release of the *T. nigroviridis *genome, the tnPKR genes were found on chromosome 17 contig (TETRAODON7:17:1829792:5098209). The 3' terminal exons of PKR1 and 5' terminal exons of PKR2 as well as middle exons of PKR3 were still missing in this contig. In this contig we identified the transcription start site of TnPKR3 to be approximately 6.6 kb downstream of the last exon of PKR2. Thus, the three TnPKR genes are tandemly arranged in a head-to-tail orientation (Figure 4).

In order to determine the genomic organization of zebrafish PKR, we searched the zebrafish genome at the Ensembl website [44] with the cloned DrPKR cDNA sequence. The first (5' region) 1409 bp of the ORF were detected in scaffold2654.7 (annotated chromosome: 18), while the 3' region was found in scaffold1952 (annotated chromosome: 18). The complete PKR ORF could be identified in these contigs. The genomic organization of DrPKR is very similar to that of tnPKR1. Both are composed of 19 exons. In both genes dsRBD2 and dsRBD3 are linked by one exon, while dsRBD3 and the kinase domain are linked by five exons.

Exons 1–14 were identified on scaffold2654.7 and exons 15–19 in scaffold1952. Long dinucleotide repeats are present 5' to exon 14 and 3' to exon 15 that might have mixed up the annotation process. Adjacent to them are stretches of 951 bp that show 99% sequence identity. Thus, it is likely that both contigs represent the same chromosomal location. We tried in vain to amplify the segment linking exons 14 and 15 by long-range PCR from genomic DNA using various parameters including different primers, annealing temperatures and polymerases.

Remarkably, PKZ was identified around 8 kb 3' to DrPKR in scaffold1952 in a head-to-tail orientation (Figure 4). The genomic organization of PKZ based on the sequence in scaffold1952 is shown in Figure 7. The organization of the kinase domains is remarkably similar to that of the PKR genes with exception that the kinase insert in PKZ is encoded by four exons (exons 4 to 7) instead of two as in the PKR genes. The kinase domain in all PKR and PKZ genes is encoded by seven exons, two exons 5' of the kinase insert and five exons 3' to it. Moreover the first, fifth and sixth exon of the kinase domain in zebrafish PKR and PKZ have exactly the same length of 120, 102 and 63 bp, respectively.

The most striking difference is that both Z-DNA binding domains of PKZ are composed of a single exon as compared with PKR where each dsRBD is encoded by two exons. There are some discrepancies between the genomic organization of PKZ as depicted in Figure 7 and the version published earlier, which was based on PCRs from genomic DNA, partial sequencing and the genomic sequences available at the time [19]. These discrepancies could be explained by contamination of the PCRs with cloned plasmid PKZ and by errors in earlier draft genomic sequences. However, the most important features, including a single exon encoding the two Zα domains, the boundaries of the exons 5 and 6, which are spliced out in one splice variant, and the length and position of a retained intron in other splice variants are concordant.

*G. aculeatus *PKR1 is organized in a similar manner to the other PKR genes. As in hsPKR, the two dsRBDs are not separated by an additional exon. Interestingly, the small linker between dsRBD2 and the kinase domain is encoded by a single exon, as in drPKZ, and as opposed to five exons in TnPKR1 and DrPKR.

### Both zebrafish PKR and PKZ phosphorylate yeast eIF2α

**Figure 8:**
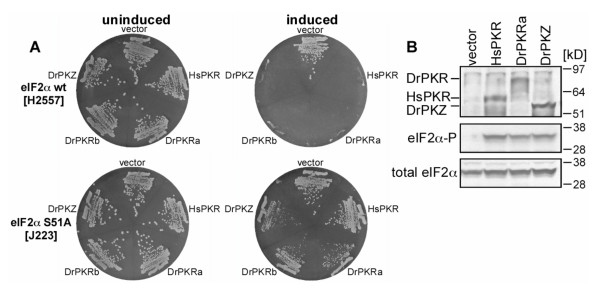
**Both zebrafish PKR and PKZ phosphorylate yeast eIF2α**. (A) Plasmids expressing full-length human (hs) PKR or zebrafish (dr) PKZ or two different alleles of PKR, under the control of a galactose-inducible promoter, were introduced into *S. cerevisiae *strains H2557 (wild-type eIF2α, upper plates) and J223 (eIF2α-S51A, lower plates) as indicated. After two rounds of single colony purification, the transformants were streaked out simultaneously on SD-ura (non-inducing conditions, left) or SGal-ura (inducing conditions, right) medium and grown for three days (SD-ura) or four days (SGal-ura). (B) Western blot analyses of extracts from strain H2557 transformed with vector, HsPKR, DrPKR or DrPKZ. SDS-PAGE was used to separate 4 μg of WCEs which were blotted onto nitrocellulose membranes. Tagged PKR and PKZ were detected using anti-Flag-tag antibody (upper panel). eIF2α phosphorylated on Ser51 and total eIF2α are shown in middle and bottom panels, respectively.

The budding yeast *S. cerevisiae *has been used to characterize vertebrate eIF2α kinases [48]. To test whether zebrafish PKR and PKZ can phosphorylate yeast eIF2α, we sub-cloned these genes into a yeast expression vector containing a galactose inducible promoter. The resulting PKR and PKZ plasmids were introduced into isogenic yeast strains lacking the only endogenous *S. cerevisiae *eIF2α kinase GCN2, expressing either wild-type eIF2α (H2557) or non-phosphorylatable eIF2α-S51A (J223). Strains transformed with empty vector and a human PKR expression construct were used as negative and positive controls, respectively. All transformants grew well under non-inducing conditions on glucose medium (Figure 8A, left). When expression was induced on galactose medium, HsPKR, the two DrPKR variants and DrPKZ exhibited strong cytotoxicity in the strain carrying wild-type eIF2α (Figure 8A, upper right). This cytotoxicity was blocked in the eIF2α-S51A mutant strain, indicating that both zebrafish PKR isoforms and PKZ can phosphorylate yeast eIF2α.

To examine eIF2α phosphorylation in yeast directly, whole-cell extracts (WCEs) of strain H2557 transformed with the expression plasmids for HsPKR, DrPKRa and DrPKZ were prepared. Following 12 h induction on galactose medium, Western blot analyses using an eIF2α 51-phospho-specific antibody confirmed that these kinases phosphorylated eIF2α on Ser51 (Figure 8B, middle panel). No eIF2α phosphorylation was observed in cells transformed with the empty vector. The eIF2α 51-phospho-specific antibody used does not react with eIF2α-S51A in the J223 strain [49].

Western blot analyses with anti-yeast eIF2α antibodies revealed comparable levels of eIF2α (Figure 8B, lower panel). Expression of PKR and PKZ, monitored with anti-Flag tag antibody (Figure 8B, upper panel), indicated that the kinases were expressed at comparable levels and had the expected molecular masses (65.4 kD for HsPKR, 80.5 kD for DrPKR and 61.7 kD for DrPKZ). These data confirm that drPKR and drPKZ are eIF2α Ser51 kinases.

## Discussion

Although PKR-like activity had been observed in fish cells, the responsible genes eluded molecular cloning for a long time. The identification of goldfish and zebrafish eIF2α kinases (PKZ), which are most closely related to PKR, was surprising because they contain two Zα domains in the N-terminus instead of dsRBDs as found in PKR [18,19]. This raised the question of whether the Zα domains actually replaced the dsRBDs in fish or if species can be identified in which PKR and PKZ actually coexist.

We have now shown that fish and two amphibian species actually possess PKR genes. Interestingly, phylogenetic analyses of PKR and PKZ genes revealed that fish PKR genes are more closely related to fish PKZ than to PKR of the other vertebrates. This indicates that a duplication of an ancestral eIF2α kinase occurred in the fish lineage after divergence of the tetrapod lineage approximately 450 millions years ago [50] and led to the emergence of both fish PKR and PKZ. It is most likely that this kinase was an ancestral PKR gene that presumably contained two dsRBDs. The most likely scenario is that the dsRBDs of one duplicated copy were replaced by Zα domains. Since both Zα domains in PKZ are encoded by a single exon, it suggests that they might have been acquired from another cellular ZBP. So far PKZ has been only identified in fish species of the orders Cypriniformes and Salmoniformes. We could not identify it in other fish species. At present it is not clear whether PKZ is present in fish species of other orders. It might have been lost during evolution in some species or just evolved in a common ancestor of *Cypriniformes *and *Salmoniformes*.

In addition to the early duplication leading to fish PKZ and PKR genes, four independent duplication events are evident. Two of these duplications can be observed in *T. nigroviridis*: first, an early duplication that gave rise to TnPKR1 and an ancestral PKR gene and, second, duplication of the latter gene led to the emergence of TnPKR2 and TnPKR3 relatively late in the evolution of this species. The other two duplications apparently occurred sometime during amphibian evolution and can be observed for *X*. *tropicalis*. Since all of the lineage-specific duplications resulted in tandemly repeated genes that are located within 1 to 10 kb, they most likely resulted from independent tandem duplication events. This is noteworthy, because a whole genome duplication occurred early during fish evolution [50,51], which is apparently not the mechanism for the origin of the duplicated PKR genes. The observation that all tandemly duplicated PKR/PKZ genes are arranged in a head-to-tail orientation is consistent with reports obtained for mammals, where the majority of tandemly arrayed genes (68–76%) were found in this orientation [52]. The presence of the duplicated genes in the same chromosomal neighborhood might be important for similar transcriptional activation after immunostimulation. This notion is supported by the comparable strong induction of zebrafish PKR and PKZ after poly(I:C) stimulation in six out of nine investigated tissues. However, high constitutive PKR expression in brain and absence of PKR expression in gonads, where PKZ is constitutively expressed at moderate levels but not induced upon stimulation, appear to be important differences. Such expression differences might have been important in the fixation of these duplications. In addition, given the important role for PKR in host defense, the duplicated PKR genes probably provided a selective advantage by leading to the recognition of an extended spectrum of viral nucleic acid structures, including both dsRNA and Z-DNA/RNA, and maybe by altering sensitivity to viral PKR inhibitors. Fixation of these duplications indicates that they provided selective advantages.

A strikingly high number of sites at which convergent evolution is evident are found within the PKR/PKZ family. At these sites, the same amino acid substitution evolved independently. At many of these sites, non-conservative amino acid substitutions can be observed. An explanation for the observed convergent evolution and the high divergence at multiple sites is that viral inhibitors of PKR and PKZ might exert strong selective pressure on these genes. A growing number of viral PKR inhibitors have been identified [1,5]. While some of these appear to directly interfere with dsRNA-mediated PKR activation, others target the PKR domain directly. Among the best-characterized PKR antagonists are the Vaccinia virus proteins E3L and K3L, orthologs of which are found in many poxviruses. E3L and K3L are important virulence factors and are involved in determining host range [53]. E3L probably inhibits PKR activation by preventing its activation by dsRNA and forming inactive heterodimers with PKR [54,55]. In addition, E3L has been reported to bind to the PKR kinase domain directly [55,56]. K3L shares homology with the N-terminus of the PKR substrate eIF2α and acts as a pseudosubstrate [57]. Iridoviruses, which are important pathogens of lower vertebrates including fish and amphibians, possess different eIF2α-related proteins called vIF2α. The 90 N-terminal residues of vIF2α display homology to the N-terminus of eIF2α, while the C-terminus of vIF2α, which is comprised of 140–150 residues, is unique and does not share any detectable homology with other proteins [58]. It has been suggested that vIF2α acts similarly to K3L to inhibit PKR-related proteins in the infected species [58,59]. The molecular characterization of amphibian PKR and fish PKR and PKZ genes paves the way for the study of viral antagonists of these kinases.

We have previously shown that zebrafish PKZ inhibits protein expression when transfected into mammalian cells [19]. In this study, we used the budding yeast *S. cerevisiae *as another heterologous system to study the functionality of both PKZ and two PKR alleles from zebrafish. This system has been used previously to study other eIF2α kinases including human PKR [48]. Similar to human PKR, both zebrafish PKR alleles and PKZ were highly toxic when expressed in yeast. This cytotoxicity was inhibited in a yeast strain expressing the non-phosphorylatable eIF2α-S51A mutant. Furthermore, expression of human PKR and zebrafish PKR and PKZ resulted in comparable levels of eIF2α phosphorylation. These findings formally demonstrate for the first time that zebrafish PKR and PKZ are eIF2α kinases, and they underscore the usefulness of this system to further characterize these kinases and their viral inhibitors.

Dimerization of PKR, which is partly mediated by residues in the kinase domain, is essential for PKR function as well as for the other eIF2α kinases. Multiple sequence alignment of the kinase domains of PKR and PKZ genes shows that only some of these residues are highly conserved. A dimer contact that is essential for activity of human PKR is mediated by a salt bridge between residues Arg262 and Asp269 [11]. While mutation of one of the residues impaired eIF2α phosphorylation, reciprocal mutation of both residues restored kinase activity. This salt bridge has also been shown to be important for yeast GCN2 and human PERK function [46]. However, amino acids forming this salt bridge are missing in 11 out of 31 sequences. Since dimerization is probably important for the activity of all PKR and PKZ proteins, it is likely that other residues in these proteins mediate dimerization. Consistently, second-site mutations that promoted kinase domain dimerization suppressed the requirement for the salt-bridge interaction in human PKR [11].

The same is probably true for the residues mediating the contacts between PKR and its substrate eIF2α because only 3 out of 12 of these residues are highly conserved. The residues of eIF2α, on the other hand, are highly conserved between the different species, that is, 100 amino acids surrounding the phosphorylation site in eIF2α are identical between zebrafish and human and hence no evidence of co-evolution between PKR and eIF2α was observed. However, this lack of sequence conservation in PKR and PKZ is consistent with the observation that most eIF2α contacts to PKR are directed at the main chain and not the side chains of the amino acids [10].

A specific feature of eIF2α kinases from multicellular eukaryotes is the presence of a highly acidic KI linking kinase subdomains IV and V. The KI is highly divergent in length ranging from 14 amino acids in *C. familiaris *PKR to 248 residues in *Caenorhabditis briggsae *PERK [19] and it appears to be important for eIF2α phosphorylation [9,60,61]. Substitution of the Ser/Thr phosphorylation sites in the KI of human PKR by alanine in combination with a T446A mutation reduced PKR activity [9]. It has been recently shown for PERK that the autophosphorylated KI is important for the recruitment and phosphorylation of eIF2α in the cell [61]. A striking difference between PKZ and PKR proteins is the length difference of the KI between PKZ (66–80 residues) and PKR (14–34 residues). PKZ evidently acquired a longer KI after the duplication that led to the emergence of fish PKR and PKZ genes by the inclusion of two additional exons. As both zebrafish PKZ and PKR are functional eIF2α kinases (Figure 8), the significance of this difference has still to be established. However, it is intriguing that the second most abundant splice variant of zebrafish PKZ encodes for a truncated KI of 15 residues [19]. In this splice variant, the two PKZ-specific exons (exons 5 and 6) are spliced out. The longer KI in primate PKR compared with PKR from other mammals is probably a result of a duplication of the KI. This duplication comprises a perfectly conserved stretch of five amino acids and apparently occurred in the beginning of primate evolution. Lineage-specific amino acid substitutions and deletions/insertions provide also intriguing insights into the evolution of PKR genes. Two amino acid substitutions, as well as a three amino acid deletion, have been identified that are exclusively present in all eutherian sequences. This pattern indicates that all of the eutherian PKR genes descended and evolved from a single ancestral allele.

Zebrafish is the first species known in which PKZ and PKR coexist. Together with HRI, PERK and GCN2, which are also found in zebrafish, the total number of different eIF2α kinases in this species is five. Even more eIF2α kinases genes (six) are found in *X. tropicalis *and *T. nigroviridis*, where we identified three PKR genes. These species also possess HRI, PERK and GCN2 (Rothenburg, unpublished).

A better understanding of the immune system of lower vertebrates and the mechanism by which pathogens circumvent the host defense response is of considerable ecological and economical importance. Viruses of lower vertebrates are abundant worldwide and have been implicated in large losses in wild fish and amphibian populations, as well as in those raised in commercial farms [40,59,62]. We have discovered heterogeneity for both zebrafish PKR, with at least two different alleles differing in four amino acids, and as well as previously for PKZ (nine alleles) [19]. Given the critical antiviral function of PKR in mammals and the importance of allelic variation in immune system-related genes in general, we hypothesize that genetic heterogeneity in the PKR/PKZ system decreases susceptibility to viruses that directly target PKR or PKZ. It might be critical to identify the genes of the innate immune system such as PKR and PKZ for economically important species and take the genotyping into consideration for animal breeding in order to achieve high levels of heterogeneity and optimal natural resistance. Furthermore, the low level of sequence conservation between PKR/PKZ genes in different species might actually be important in limiting inter-species transmission of viruses.

## Conclusion

In this paper, we have demonstrated the presence of PKR genes in various fish and amphibian species. Independent duplication events occurred in different fish and amphibian lineages during the evolution of PKR and PKZ leading to tandemly arrayed genes. Fixation of these duplications indicates that they provided selective advantages presumably by leading to the recognition of an extended spectrum of viral nucleic acid structures and perhaps by altering sensitivity to viral PKR inhibitors. Our findings support the notion that PKR and PKZ play similar but not identical roles in host defense and open the door to the investigation of the role of PKR and PKZ in the innate immune response of poikilotherms and in the interplay with viral proteins and nucleic acids. The finding that both zebrafish PKR and PKZ phosphorylate eIF2α in yeast is an indication of the usefulness of this system for the characterization of the interaction of viral gene products with PKR and PKZ.

## Methods

### Cloning of PKR genes *T. nigroviridis*, *D. rerio *and *X. laevis*

Twelve hours after intraperitoneal injection of 10 μg/g bodyweight poly(I:C) (Sigma) for 12 h, total RNA was prepared from a *T. nigroviridis *fish, which was purchased in a local pet shop, using Trizol (Invitrogen). cDNA was prepared using SuperScript II reverse polymerase (Invitrogen) using random hexamers according to manufacturer's protocol. Cytochrome B (cytb) gene was amplified with primers described in [42] and sequenced. Sequences of primers described hereafter are shown in Table 1.

**Table 1:** primers used in this study

Primer name	Sequence (5' → 3')
tnPKR1race1R	(5'-CAAAGTCTCCGATCTTTAC-3')
tnPKR2race1R	(5'-AAAGTCCCCAATCTTCAC-3')
tnPKR1 2R	(5'-CACAGCTCCATCTGGATATAGAGG-3')
tnPKR2 2R	(5'-CTCTCCTGACTCCTCTTGGAGTC-3')
tnPKR1 3R	(5'-GGGTATTTGTGACCATCGATCG-3')
tnPKR2 3R	(5'-CTGACTCCGAACTGCCTGTGGAC-3')
tnPKR1 1F	(5'-CGGACCTCGATCACTGCAATATTATTCG-3')
tnPKR1 2F	(5'-GTGGAGTTGAGTACATCCACTCC-3')
tnPKR2 1F	(5'-CTGACTTGTTACACATCAATATTATTCG-3')
tnPKR2 2F	(5'-CTCAGCAGATAGCGAGTGGAGTG-3')
tnPKR1 3F	(5'-GAGAACGGTGTACAAAGGAACC-3')
tnPKR1 5F	(5'-CCAGAAAGTGGATATATTTCCTTTGGGC-3')
tnPKR2 3F	(5'-CTTCATCCACAGAGACCTGAAGCC-3')
tnPKR1 5'1F	(5'-CGGATTCCGCTGGAAGACACG-3')
tnPKR1 sto1R	(5'-GACAGTCTTTCTCTCATTGGGCTC-3')
tnPKR2 st1F	(5'-CCACCCTTTTATCTGCTGAATTTTACC-3')
tnPKR2 sto2R	(5'-TCGTTGTTTACCGGGTCCGAGC-3')
tnPKR3 sto1R	(5'-GGAAATACTGGCTGTCATTTGCAATAA-3')
tnPKR1 3'utr1R	(5'-GTAAAAAGGAGACACATCTCTTAACCC-3')
tnPKR2 g4R	(5'-GCCGAGGAGAGCAGACTTACC-3')
drPKR 2F	(5'-CTGCTTCGTTTTATTCGGCATTCGC-3')
drPKR 5R	(5'-ATGATCCTATAAGAGAGGAGCTGG-3')
xlPKR1 1F NheI	(5'-AAGGTAGCTAGCCATTGAGTAAGAAGCCTTTCTGATG-3')
xlPKR1 1R KpnI	(5'-TGGGGTACCATGGGTTTTTGAGTCCAAGTTG-3')

We performed 5' and 3' RACE experiments using 5' RACE and 3' RACE systems (Invitrogen) according to the manufacturer's protocol. Relative positions of primers used for these experiments are shown in Additional file 3. Briefly, for 5' RACE, 2 μg of total RNA prepared from skin was primed with tnPKR1race1R and tnPKR2race1R. Initial PCRs were performed with Abridged Anchor Primer and tnPKR1 2R or Tn PKR2 2R for 25 cycles at 95°C for 30 s, 58°C for 30 s and 72°C for 2 min. Nested PCRs were performed using Abridged Universal Anchor Primer and tnPKR1 3R and tnPKR2 3R using identical cycling conditions as noted above. Reactions were performed using AmpliTaq Gold polymerase (PE Biosystems) and initiated with a heat activation step at 95°C for 4 min. For 3' RACE, RNA was primed with Adapter Primer. Initial PCRs were performed with primers tnPKR1 1F (reaction a), tnPKR1 2F (reaction b), tnPKR2 1F (reaction c) and tnPKR2 2F (reaction d) for 25 cycles at 95°C for 30 s, 60°C for 30 s and 72°C for 3 min. Nested PCRs were performed with Abridged Universal Anchor Primer using tnPKR1 3F and tnPKR1 5F with reaction a (Figure 1B, lanes 1 and 2) and reaction b (Figure 1B, lanes 3 and 4) as templates, respectively. Reaction c was used with primers tnPKR2 2F and tnPKR2 3F (Figure 1B, lanes 5 and 6), respectively. Reaction d was used with forward primer tnPKR2 3F (Figure 1B, lane 7). Identical PCR cycling conditions were used as in the initial PCRs. Complete ORFs were amplified using tnPKR1 5'1F × tnPKR1 sto1R (for PKR1), tnPKR2 st1F × tnPKR2 sto2R (for PKR2) and tnPKR2 st1F tnPKR3 sto1R (for PKR3) using Platinum Taq DNA Polymerase High Fidelity (Invitrogen) and the following PCR conditions: 2 min at 95°C followed by 34 cycles at 95°C for 30 s, 60°C for 30 s and 72°C for 3 min. PCR with genomic DNA from *T. nigroviridis *skeletal muscle was performed with Platinum Taq DNA Polymerase High Fidelity (Invitrogen) at 94°C for 2 min followed by 36 cycles of 95°C for 30 s, 60°C for 30 s and 72°C for 8 min. All PCR products were cloned using TOPO-TA cloning kit (Invitrogen) and completely sequenced.

PCR spanning the 5' untranslated region of PKR1 and exon 15 of PKR1 was performed with primers tnPKR1 5'1F × tnPKR1 2R. The region spanning exon 14 and the 3' untranslated region of exon 19 of PKR1 was amplified with primers tnPKR1 1F × tnPKR1 3'utr 1R. Primers tnPKR1 5F (in exon 17 of PKR1) and tnPKR2 g4R (in exon 7 of PKR2) were used to amplify the intergenic region between PKR1 and PKR2. After cloning into the TA-Topo vector, all inserts were completely sequenced using walking primers.

The complete ORF of *D. rerio *PKR was amplified using Platinum Taq with primers drPKR 2F and drPKR 5R for 95°C for 2 min followed by 32 cycles of 95°C for 30 s, 60°C for 30 s, 72°C for 2.5 min). Expression analysis was performed with cDNA from poly(I:C) and PBS (control) treated zebrafish as described previously [19]. PCRs for PKZ and RACK1 were performed on the same day as amplification of PKR as described [19].

cDNA from *X. laevis *spleen was PCR amplified using KOD polymerase (Novagen) with primers xlPKR1 1F NheI and xlPKR1 1R for 2 min at 95°C followed by 32 cycles at 95°C for 30 s, 55°C for 30 s and 72°C for 2 min, digested with NheI and KpnI and directly cloned into pcDNA3.1 myc-his (Invitrogen) and completely sequenced.

### Yeast strains and plasmids

Zebrafish PKR and PKZ as well as human PKR were sub-cloned into the yeast expression vector pYX113 (R&D systems) containing the *GAL-CYC1 *hybrid promoter and the selectable marker *URA3*. The vector was modified to contain a Precision protease target site and His(6) and Flag tags at the 3' end of the multiple cloning site. Standard methods were used for culturing and transforming yeast strains. Yeast strains H2557 (*MATαura3-52 leu2-3 leu2-112 gcn2Δ*) and J223, a derivative of H2557 in which wild-type *SUI2 *(eIF2α) was replaced with *SUI2-S51A *[11], were transformed with the expression plasmids using the LiAcetate/PEG transformation method and grown on SD-ura plates. For each transformation, four single colonies were picked and colony purified. Colonies were streaked out in parallel on SD minus uracil (non-inducing conditions) and Sgal minus uracil medium (containing 10% galactose and all amino acids except uracil; inducing conditions) and grown at 30°C for 3–4 days.

### Immunoblot analysis

For immunoblot analysis, yeast transformants were grown overnight in SD + trp medium. Saturated cultures were diluted to 1:35 in synthetic complete medium minus uracil (SC-Ura, containing 2% dextrose and all amino acids except uracil) and grown for 6 h at 30°C (OD_600 _~ 0.8–1.0). Cells were harvested by centrifugation, resuspended in SGal minus uracil medium (Sgal-Ura, containing 10% galactose and all amino acids except uracil) and grown at 30°C for 12 h to induce PKR/PKZ expression. Cells were harvested, washed with ice-cold water, resuspended in breaking buffer (40 mM Tris-HCl pH 7.4, 15 mM EDTA, 1 mM PMSF, 45 mM NaF, 1 μM 2-aminopurine, 25 mM β-glycerophosphate, 120 mM (NH4)_2_SO_4_, 1 mM DTT and one protease inhibitor tablet (Roche diagnostics) per 10 ml buffer) and broken using glass beads by continuous vortexing for 15 min at 4°C. WCEs were clarified by centrifugation at 13,000 rpm for 20 min. SDS-PAGE was used to separate 4 μg of WCEs. Proteins were transferred to nitrocellulose membranes at 20 V for 2 h at 4°C and probed with rabbit phosphospecific antibodies directed against phosphorylated Ser51 of eIF2α (BioSource International). The membrane was stripped and probed with polyclonal antibodies against eIF2α (see [63]). Flag-tagged proteins were detected with anti-Flag Antibody (Applied Biological Materials).

### Phylogenetic analyses

The amino acid sequences of PKR, PKZ and PERK kinase domains (see Table 2 for accession numbers) were aligned using MUSCLE, a multiple sequence alignment software [64], and the resulting alignments were checked using MacClade (Sinauer Associates). The phylogenetic relationships within the extended PKR/PKZ family were analyzed with the amino acid sequences of the kinase domains, excluding kinase inserts between β-sheets 4 and 5, which are highly variable in size and sequence, thus not permitting expedient alignments. Phylogenetic analyses were performed using maximum likelihood [65] with nodal support assessed via bootstrapping (1000 pseudoreplicates) [66] as implemented in PHYML [67] and Bayesian methods [68] coupled with Markov chain Monte Carlo (BMCMC) inference as implemented in MrBayes v3.1.2 [69]. Model selection for these analyses was performed using ProtTest [70]. The best model chosen for this data set was JTT+I+G+F, selected by the Akaike information criterion (AIC). For the BMCMC techniques, two independent analyses were run each consisting of four chains. Each Markov chain started from a random tree and ran for 10^7 ^cycles, sampling every 1000th generation. In order to confirm that our Bayesian analyses converged and mixed well, we monitored the fluctuating value of likelihood and compared means and variances of all likelihood parameters and likelihood scores from the independent runs using the program Tracer v1.2.1 [71]. The two independent BMCMC runs resulted in identical results. These phylogenetic analyses were performed on the supercomputing cluster of the College of Life Sciences at Brigham Young University.

**Table 2:** Accession numbers of genes used for phylogenetic analyses

Species (common name)	Gene	Accession number
*Bos taurus *(cattle)	PKR	AB104655
*Canis familiaris *(dog)	PKR	NM_001048135
*Carassius auratus *(goldfish)	PKZ	AY293929
*Chlorocebus aethiops *(African green monkey)	PKR	AY623897
*Danio rerio *(zebrafish)	PKR	AM421526
*Danio rerio *(zebrafish)	PKZ	AJ852018
*Danio rerio *(zebrafish)	PERK	XM_695386
*Equus caballus *(horse)	PKR	AY850106
*Gallus gallus *(chicken)	PKR	AB125660
*Gasterosteus aculeatus *(three spined stickleback)	PKR1	AM850085
*Homo sapiens *(human)	PKR	NM_002759
*Homo sapiens *(human)	PERK	AF193339
*Macaca mulatta *(Rhesus macaque)	PKR	EF467667*
*Mesocricetus auratus *(golden hamster)	PKR	DQ645944
*Monodelphis domestica *(gray short-tailed opossum)	PKR	ENSMODT00000030492**
*Mus musculus *(house mouse)	PKR	NM_011163
*Ornithorhynchus anatinus *(platypus)	PKR	ENSOANT00000006231**
*Oryctolagus cuniculus *(European rabbit)	PKR	DQ115394
*Oryzias latipes *(Japanese medaka)	PKR	AM850088
*Pan troglodytes *(chimpanzee)	PKR	XM_001166391
*Pimephales promelas *(fathead minnow)	PKR	AM850089
*Rattus norvegicus *(Norway rat)	PKR	NM_019335
*Salmo salar *(Atlantic salmon)	PKZ	ABA64562
*Sus scrofa *(pig)	PKR	NM_214319
*Takifugu rubripes *(torafugu)	PKR1	AM850086
*Takifugu rubripes *(torafugu)	PKR2	AM850087
*Tetraodon nigroviridis *(green spotted pufferfish)	PKR1	AM421523
*Tetraodon nigroviridis *(green spotted pufferfish)	PKR2	AM421524
*Tetraodon nigroviridis *(green spotted pufferfish)	PKR3	AM421525
*Xenopus laevis *(African clawed frog)	PKR1	AM421528
*Xenopus tropicalis *(western clawed frog)	PKR1	AM850090
*Xenopus tropicalis *(western clawed frog)	PKR2	AM850091
*Xenopus tropicalis *(western clawed frog)	PKR3	AM850092

* For phylogenetic analyses a *Macaca mulatta *PKR sequence was used that is identical to the deduced amino acid sequence EF467667, except for an Asn to Arg substitution at position 384.

** Ensembl identification numbers are shown.

### Databases

The most current database releases at NCBI [72] and Ensembl [44] (genomic and ESTs databases, using BLASTN and TBLASTN) were searched for PKR and PKZ genes using various known PKR and PKZ sequences as probes.

### Data deposition

The sequences reported in this paper have been deposited with the EMBL Nucleotide Sequence Database: *T. nigroviridis *PKR1 (genomic) [EMBL:AM850084]; *T. nigroviridis *PKR1 [EMBL:AM421523]; *T. nigroviridis *PKR2 [EMBL:AM421524]; *T. nigroviridis *PKR3 [EMBL:AM421525]; *D. rerio *PKR allele a [EMBL:AM421526]; *D. rerio *PKR allele b [EMBL:AM421527]; *G. aculeatus *PKR1 [EMBL:AM850085]; *T. rubripes *PKR1 [EMBL:AM850086]; *T. rubripes *PKR2 [EMBL:AM850087]; *O. latipes *PKR [EMBL:AM850088]; *P. promelas *PKR [EMBL:AM850089]; *X. laevis *PKR1 [EMBL:AM421528]; *X. tropicalis *PKR1 [EMBL:AM850090]; *X. tropicalis *PKR2 [EMBL:AM850091]; *X. tropicalis *PKR3 [EMBL:AM850092].

## Authors' contributions

SR and ND performed cloning, expression analyses and database searches. LT and SR performed sequence alignments and phylogenetic analyses. MD and SR carried out yeast experiments. SR prepared the figures and wrote the paper with contributions from all authors. The study was conceived and supervised by SR with important contributions from TED.

## Supplementary Material

Additional file 1 Multiple sequence alignment of PKR double-stranded RNA binding domains. Secondary structure elements as reported for human PKR (Nanduri et al., 2000) are shown above the sequences. Background of residues that are highly conserved are colored as follows: 100% conservation = dark green; ≥90% conservation = light green; ≥80% conservation = yellow; conservation of functionally conserved residues = salmon. Consensus sequences using weblogo [73] are indicated above the alignment. Click here for file

Additional file 2 Multiple sequence alignment of the kinases inserts linking PKR and PKZ β4 and β5. Acidic residues are highlighted in blue (Asp, D) and azure (Glu, E). Ser (S) and Thr (T) residues are highlighted in red and orange respectively. A perfectly conserved 5 amino acid duplication, observed for the primate kinase inserts, is boxed. DrPKZs denotes the short splice variant of zebrafish PKZ in which exons 5 and 6 are spliced out. Click here for file

Additional file 3 Relative positions of primers used for 5' and 3' rapid amplification of cDNA ends of *T. nigroviridis *PKR genes. Position of primers used for 5' RACE experiments are indicated above the genes, primers used for 3' RACE experiments are shown below the genes. PCR products shown in Fig. 1B are schematically indicated by dashed lines. Domains are shown in the following colors: dsRNA binding domains (dsR): blue; kinase domains (KD): red; kinase inserts (KI): yellow. Click here for file
