# Supplementary material for: Double-stranded RNA-activated protein kinase PKR of fishes and amphibians: Varying the number of double-stranded RNA binding domains and lineage-specific duplications
Source: BMC Biol. 2008 Mar 3;6:12. doi: 10.1186/1741-7007-6-12 (PMC2291453; doi:10.1186/1741-7007-6-12)
Supplement: Additional file 1 — Multiple sequence alignment of PKR double-stranded RNA binding domains. Secondary structure elements as reported for human PKR (Nanduri et al., 2000) are shown above the sequences. Background of residues that are highly conserved are colored as follows: 100% conservation = dark green; ≥90% conservation = light green; ≥80% conservation = yellow; conservation of functionally conserved residues = salmon. Consensus sequences using weblogo [73] are indicated above the alignment. [file 1741-7007-6-12-S1.pdf]

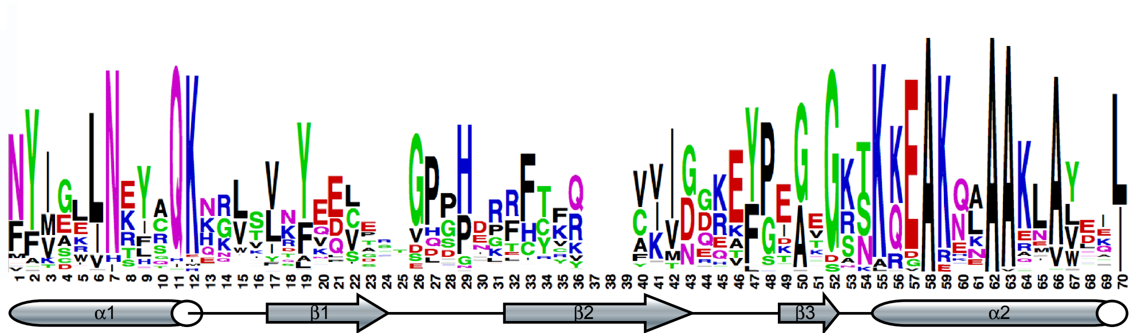


```
HsPKR dsRB1 FFMEELNTYRQKQGVVLKYQELPNSGPPHDRRFTFQ---VIIDGREFPEGEGRSKKEAKNAAAKLAVEIL
PtPKR dsRB1 FFMEELNTYRQKQGVVLKYQELPNSGPPHDRRFTFR---VIIDGREFPEGEGRSKKEAKNAAAKLAVEIL
CaePKRdsRB1 FYMEELNKYRQKQGVTFSYQELPNTGPPHDRRFTFR---VVTDRREFPEAEGRSKKEAKNAAAKLAVDIL
MamPKRdsRB1 FYMEELNKYRQKQGISLSYQELPNTGPPHDRRFTFQ---VVTDRRTFPEAEGRSKKEAKNAAAKLAVDIL
CfPKR dsRB1 FFIEELNIFCQKHRKVLKYNELSKEGPAHNLRFTFQ---AIIDEREYPEAEGKSKKEARNTAAKLALEIL
EcPKR dsRB1 VFIEELNKYRQKHNVVLDFRELSKTGPPHDLTFTFR---VIINGREFPEAEGKSKKEAKNAAAKMAVEIL
BtPKR dsRB1 FYIEELNKYQQKNGVEVRYCELAKTGPPHNFRFTYQ---VIIDDKEYPKAEGRSKKEAKNAAAKLALEII
SsPKR dsRB1 FYIEELNKYQQKNDVILKYRELCKTGPAHNLRFTYQ---VVIDEKEFPKAEGRSKKEAKNAAVKLAFEII
OcPKR dsRB1 FFIEELNKYRQKHSILLKYHELPQSGPPHDVTFTFK---VIINEREFPEAEGKSKKEAKNAAAKIAVDIL
MaPKR dsRB1 MYMDKLNKYHQKHRVTITYKHLFTTGPPHDRRFTFQ---VIIDGEEFPEAEGKTKQEAKNAAAKLAVDTL
MmPKR dsRB1 FYMDKLNKYRQMHGVAITYKELSTSGPPHDRRFTFQ---VLIDEKEFPEAKGRSKQEARNAAAKLAVDIL
RnPKR dsRB1 FYVDKLNKYSQIHKVKIIYKEISVTGPPHDRRFTFQ---VIIEEREFPEGEGRSKQEAKNNAAKLAVEIL
GgPKR dsRB1 ECMAKINRYCQKNKYKLDYVDVDMRGPSHDPEFEVV---VKINGVKYGTGVGKSKKEAKAAAAAKTWDMI
XlPKR dsRB1 NVKGQLISFCTKNGLSYQFKKVEATGPSHDPRFTFQ---VFVNGEKLGEGQDKKKKGAECMAAKMALSTL
XtPKR dsRB1 NAKGQLITFCIKNGLAYHFNEVEATGPSHDPRFTSQ---VFVNQEKLGEGQDKKKKGAENIAAKMALLTL
TnPKR1dsRB1 NYISLLNEQAQKEGWSLRYEDVGCDGPDHFRTFKIV---TIINDRAFPEGVGKNKKDAKQKAAENAWTAL
TnPKR2dsRB1 NYISLLNEQAQKQGWSLRYEDVGCDGPDHIKRFRVR---AIVNGRAFPEGAGKNKRDAKHKAAENAWTAL
TrPKR1dsRB1 NYVARLNEYAQENRLSLQYQDVGCDGPDHLKTFRVR---AVIDDQAYPEGLGKNKKEAKKKAAENAWRAL
TrPKR2dsRB1 NYLIILNEHTQKMRWKLQFEDVGCDGPDHNKVFRVR---AVIDDQAYPEGLGKNKKEAKKKAAEIAWRAL
GaPKR dsRB1 MAVARLNELTQKQRLVVRYEDVGSVGPDHIKTFTVR---AVVDGKPYPDGVGKNKREAKLNAAANALTVL
DrPKR dsRB1 NYTSLLNEYQQKTQCTVEFEEGPTDGPSHNKRFTMR---AIVNGQKFPDGTGKTKKEAKQNAAKNALEGL
HsPKR dsRB2 NYIGLINRIAQKKRLTVNYEQCASGVHGPEGFHYK----CKMGQKEYSIGTGSTKQEAKQLAAKLAYLQI
PtPKR dsRB2 NYIGLINRIAQKKRLTVNYEQCASGVHGPEGFHYK----CKMGQKEYSIGTSSTKQEAKQLAAKLAYLQI
CaePKRdsRB2 NYIGLINRIAQKKRLTVNYEQCASGVHGPEGFHYK----CKMGQKEYSIGTSSTKQEAKQLAAKLAYLQI
MamPKRdsRB2 NYIGVVNRIAQKKRLTVNYEQCTSGVHGPEGFHYK----CKIGDKEYGIGTGSSKQEAKQLAAKLALLEI
CfPKR dsRB2 NYIGRMNRLAQKEQLSVNYEQWELKDCGPERFHCR----CKIGQKEYDIGKGATKQEAKHLAAKFAYEQI
EcPKR dsRB2 NYIGLINTYTQRNQLSLNYEQYESRDGGPKRFRCK----YKIGWKEYPIASGSTKQEAKQLAAKLAYDQI
BtPKR dsRB2 NYIGRLNTISQKKNLCVTYEECKSKGDGPEGFHYI----CKIGQEEYGSGVGSTKQEAKQLAAKLAYEKI
SsPKR dsRB2 NYIGRINTISQKKNLSVNYEPCEPGEDGPEKFHYK----CKIGRKVYGSGVGSTKQEAKQLAAKQAYEKL
OcPKR dsRB2 NYIGLINRVAQKAKLTVNYEPCELWEQGPKRFQYK----CRIGLKVYGTGIGSTKQEAKQLAAKSAYHKI
MaPKR dsRB2 NYIGLVNSYAQKEKLSVNYQQCAFNTQSPQRFCYK----CIIGLKTYGIGSGATKQEAKQLAAKEAYQKL
MmPKR dsRB2 NYIGLVNSFAQKKKLSVNYEQCEPNSELPQRFICK----CKIGQTMYGTGSGVTKQEAKQLAAKEAYQKL
RnPKR dsRB2 NYIGLVNSFAQKENLPVNFELCDPDSQLPHRFICK----CKIGQTTYGTGFGANKKEAKQLAAKNAYQKL
GgPKR dsRB2 DYISLLNKYSQKTLQLVDYNNINRTGDPHAPMYSCS---CVISGHVYGNGKGNSLAVAKQAAAKEAYEVL
XlPKR dsRB2 NYVGILHELCQKHTLIVTFLD-ERHGQPHIPEFFCK---AVIGKEEFPKAKGKNKKEAKRKAAHLALISL
XtPKR1dsRB2 NYVGKLHELCQKHKLICKFFD-ECYGLPHIPEFWCK---VVIGEEEFHKVKGNNKKEAKRKAAYFALMSL
TnPKR1dsRB2 KFVCWLNEYGHKNWVKVKPVESTRVSPQWTGPCCR----FVVGDKEYPEAVGKTKREAKEEAARLVYNEI
TrPKR1dsRB2 NFVAWLNEYGQKNRVSVKLVESTRPGLRGAELCCR----FVVGDQEYPDAVGKTKREAKEEAARLVYNEI
TrPKR2dsRB2 NYVAWLNEYGQKNRVSVKLVESTRPGLRGAELCCR----FVVGDQEYPDAVGKTKREAKEEAARLVYNEI
GaPKR dsRB2 NYMCWLNEYSQKNRMMIRAVESTQLVPNGLSLCCCY---FVVGDKEYPRATGKTKKEAKEEAAKLVFQQI
DrPKR dsRB2 NYTCWLNEHSQKSRLMFKACESTKMDPGNLTRLCTYVCKYVCDDKEFPEGYGKNKKEAKEAAALRVYEEL
TnPKR1dsRB3 NYISLINEHCQKKGFSHSFVMVDRQGPSHGPQFYYQ---LSIDGHKYPVGEGKTAKEARQNAAQLAWPVL
DrPKR dsRB3 NYIAYLNNYCQKKKRVYDFKLVDRIGPPHNPIFVYK---VVMDGKEYPEAQGRNAKEAKQNAAQHAWSEI
```

Additional Figure 1.

Multiple sequence alignment of PKR double-stranded RNA binding domains
Secondary structure elements as reported for human PKR (Nanduri et al., 2000) are shown above the sequences. Background of residues that are highly conserved are colored as follows: 100% conservation = dark green; ≥90% conservation = light green; ≥80% conservation = yellow; conservation of functionally conserved residues = salmon pink.
Consensus sequences using weblogo (http://weblogo.berkeley.edu/) are indicated above the alignment.
