# Supplementary material for: Double-stranded RNA-activated protein kinase PKR of fishes and amphibians: Varying the number of double-stranded RNA binding domains and lineage-specific duplications
Source: BMC Biol. 2008 Mar 3;6:12. doi: 10.1186/1741-7007-6-12 (PMC2291453; doi:10.1186/1741-7007-6-12)
Supplement: Additional file 2 — Multiple sequence alignment of the kinases inserts linking PKR and PKZ β4 and β5. Acidic residues are highlighted in blue (Asp, D) and azure (Glu, E). Ser (S) and Thr (T) residues are highlighted in red and orange respectively. A perfectly conserved 5 amino acid duplication, observed for the primate kinase inserts, is boxed. DrPKZs denotes the short splice variant of zebrafish PKZ in which exons 5 and 6 are spliced out. [file 1741-7007-6-12-S2.pdf]

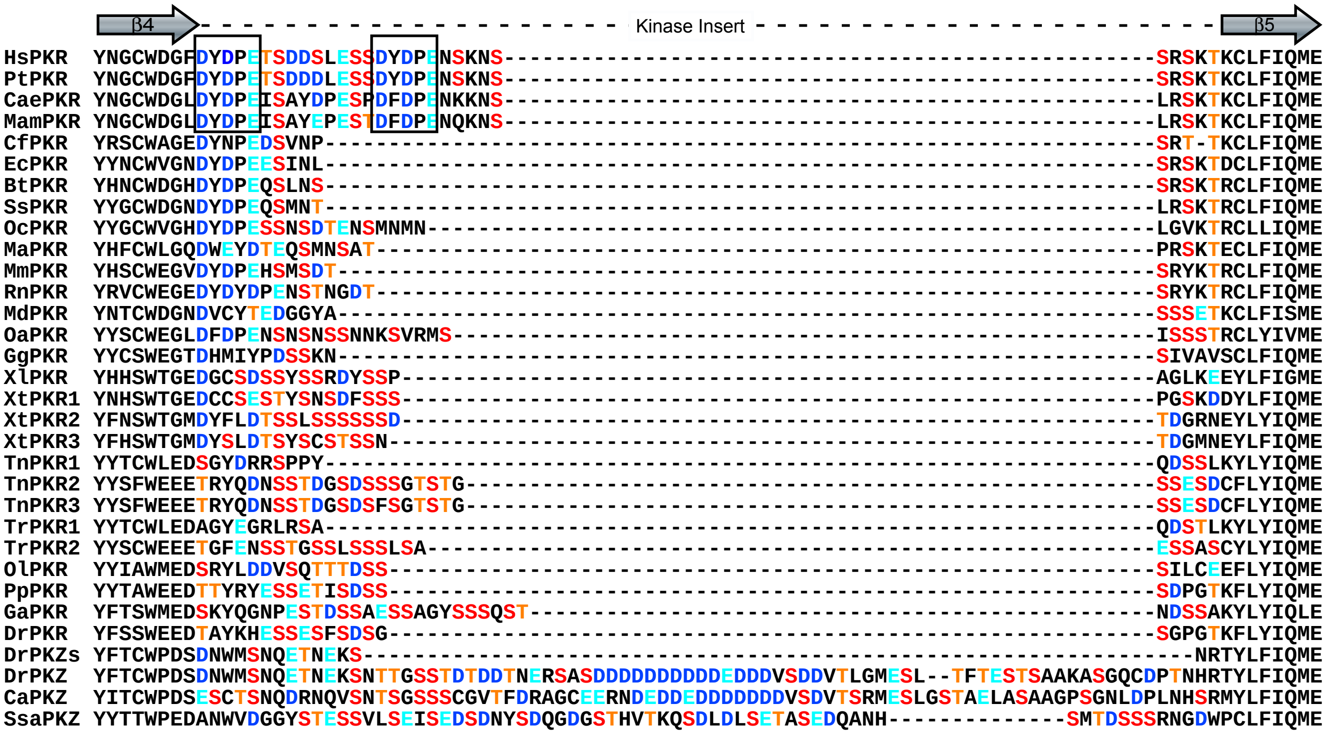


**Additional Figure 2.**

Multiple sequence alignment of the kinases inserts linking PKR and PKZ β4 and β5.
Acidic residues are highlighted in blue (Asp, D) and azure (Glu, E). Ser (S) and Thr (T) residues are highlighted in red and orange respectively. A perfectly conserved 5 amino acid duplication, observed for the primate kinase inserts, is boxed. DrPKZs denotes the short splice variant of zebrafish PKZ in which exons 5 and 6 are spliced out.
