# Supplementary material for: Double-stranded RNA-activated protein kinase PKR of fishes and amphibians: Varying the number of double-stranded RNA binding domains and lineage-specific duplications
Source: BMC Biol. 2008 Mar 3;6:12. doi: 10.1186/1741-7007-6-12 (PMC2291453; doi:10.1186/1741-7007-6-12)
Supplement: Additional file 3 — Relative positions of primers used for 5' and 3' rapid amplification of cDNA ends of T. nigroviridis PKR genes. Position of primers used for 5' RACE experiments are indicated above the genes, primers used for 3' RACE experiments are shown below the genes. PCR products shown in Fig. 1B are schematically indicated by dashed lines. Domains are shown in the following colors: dsRNA binding domains (dsR): blue; kinase domains (KD): red; kinase inserts (KI): yellow. [file 1741-7007-6-12-S3.pdf]

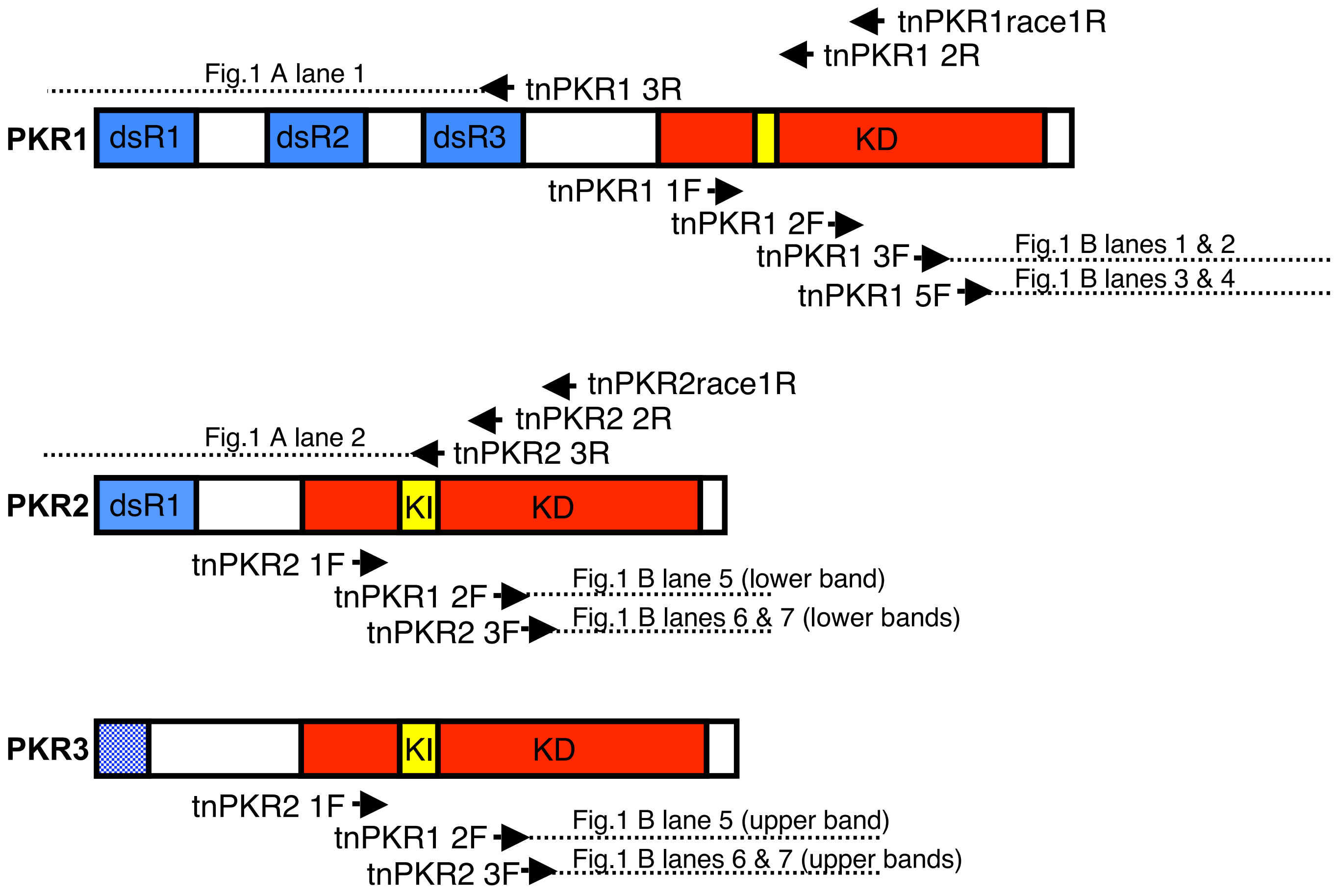

Additional Figure 3.
Relative positions of primers used for 5' and 3' rapid amplification of cDNA ends of *T. nigroviridis* PKR genes.
Position of primers used for 5’ RACE experiments are indicated above the genes, primers used for 3’ RACE experiments are shown below the genes. PCR products shown in Fig. 1 B are schematically indicated by dashed lines.
Domains are shown in the following colors: dsRNA binding domains (dsR): blue; kinase domains (KD): red; kinase inserts (KI): yellow.
